# Inactivation of the p15 gene in children with acute lymphoblastic leukemia

**DOI:** 10.1590/S1516-31802003000500005

**Published:** 2003-09-01

**Authors:** Rosana Cipolotti, José Alexandre Rodrigues Lemos, Ricardo Defavery, Carlos Alberto Scrideli, Amaury Lellis Dal Fabbro, Luiz Gonzaga Tone

**Keywords:** Acute lymphoblastic leukemia, Lymphoblastic leukemia, Tumor suppressor gene, p15, Leucemia linfóide, Genes supressores de tumor, p15

## Abstract

**CONTEXT:**

Tumor suppressor genes act on the control of cell cycle progression. In pediatric neoplasias, some of these genes may be considered to be markers for diagnosis or relapse, thus probably representing prognostic indicators.

**OBJECTIVE:**

To study the inactivation of the p15 gene in children with acute lymphoblastic leukemia.

**TYPE OF STUDY:**

Retrospective study.

**SETTING:**

Laboratory of Molecular Biology, Department of Pediatrics, Faculdade de Medicina de Ribeirão Preto, Universidade de São Paulo.

**PARTICIPANTS:**

Eighty-three children and adolescents with acute lymphoblastic leukemia were studied, with the examination of 83 bone marrow samples obtained at diagnosis, four obtained also during relapse, and two cerebrospinal fluid samples obtained from two cases of isolated relapse in the central nervous system.

**MAIN MEASUREMENTS:**

Homologous deletion of the p15 gene by multiplex polymerase chain reaction, and screening for point mutations by polymerase chain reaction/single-strand conformational polymorphism.

**RESULTS:**

Deletion of exon 2 of the p15 gene was observed in 15 children, including one case in which deletion was only verified during isolated central nervous system relapse. No case of exon 1 deletion, or that was suggestive of point mutations, was observed and no association between p15 gene inactivation and classic risk factors was established.

**CONCLUSION:**

According to the literature, inactivation of the p15 gene by deletion of exon 2 in acute lymphoblastic leukemia found in the population studied would be considered to be a molecular marker for diagnosis or relapse. However, no correlation between p15 gene deletion and clinical prognostic indicators was observed.

## RESUMO

**CONTEXTO:**

Os genes supressores tumorais atuam no controle da progressão do ciclo celular. Em neoplasias pediátricas, alguns destes genes podem ser considerados marcadores de diagnóstico ou de recaída, sendo, portanto, prováveis indicadores prognósticos.

**OBJETIVO:**

Estudar a inativação do gene p15 em crianças com leucemia linfóide aguda.

**TIPO DE ESTUDO:**

Estudo retrospectivo.

**LOCAL:**

Laboratório de Biologia Molecular, Departamento de Puericultura e Pediatria, Faculdade de Medicina de Ribeirão Preto, Universidade de São Paulo.

**PACIENTES:**

Foram estudadas 83 crianças e adolescentes com leucemia linfóide aguda, sendo examinadas 83 amostras de medula óssea ao diagnóstico, quatro também na recaída e duas amostras de líquido cefalorraquidiano em dois casos de recaída isolada em sistema nervoso central.

**VARIÁVEIS ESTUDADAS:**

Deleção homóloga do gene p15, por reação em cadeia de polimerase-*multiplex* e triagem para mutação de ponto por reação em cadeia de polimerase-polimorfismo conformacional em fita simples.

**RESULTADOS:**

Deleção do exon 2 do gene p15 foi observada em 15 crianças, incluindo um caso apenas na recaída isolada em sistema nervoso central. Nenhum caso de deleção do exon 1 foi verificado, bem como nenhum caso sugestivo de mutação de ponto. Não foi estabelecida associação entre inativação do gene p15 e fatores de riscos clássicos.

**CONCLUSÃO:**

De acordo com a literatura, a inativação do gene p15 por deleção do exon 2 em leucemia linfóide aguda encontrada na população estudada pode ser considerada um marcador molecular de diagnóstico ou de recaída. No entanto, não foi observada qualquer correlação entre deleção do gene p15 e indicadores clínicos prognósticos.

## INTRODUCTION

Childhood cancer can be distinguished from other cancers by its short latency period, rapid growth and invasive aggressiveness, as well as by an apparent reduced influence from environmental carcinogens. These peculiarities apply to the most frequent type of childhood cancer, i.e. acute lymphoblastic leukemia.^1^ Studies carried out in the 1960s showing that the fusion of normal and neoplastic cells can suppress tumorigenicity led to the isolation of the Rb tumor suppressor gene in 1986.^2^

The cell cycle consists of an alternation between periods of rest and periods of cell division until cell death. Although the duration of the different phases varies according to cell type, at least two great barriers, in the G1→S and G2→M transitions, seem to operate in all eukaryotic cells. These cells only divide after receiving instructions or extracellular stimulation, either in the form of stimulating circulating mitogenic substances, or blockers of antiproliferative cytokines, or even through contact with adjacent cells or substrates. The transition from the early/intermediate phase to the late G1 phase is called the restriction point (R point), while transitions that occur at other points of the cell cycle are called checkpoints, with the most important at the G2 to M transition. Cancerous cells abandon their control mechanisms and continue to divide without evolving to programmed death.^2^ The decision to divide occurs as soon as the cell passes the R point, with the cell following its own program thereafter until division. The passage of a cell through the R point and checkpoints is regulated by a family of kinase proteins that include a regulatory subunit, cyclin, and a catalytic subunit, cyclin-dependent kinase (CDK). Cyclin D activates CDK4, and also CDK6 in some cells, thus leading the cell to go beyond the R point.^3^

The genes p16, p15 and ARF1/p19 of the *INK4* family are a group of cyclin inhibitors located on the 9p21 band, a frequent site of abnormalities in human tumors, with p15 and p16 forming the multiple tumor suppressor (MTS) locus.^4^ The products of these genes bind to CDK 4 and 6, thus inhibiting the CDK-cyclin D complex, which results in the blockade of the phosphorylation of protein Rb, thereby preventing cell cycle progression. Genes with this mode of action are called tumor suppressor genes and have been studied as risk indicators for poor therapeutic response. Some studies^5,6^ have found an increased rate of p15 inactivation in children with acute lymphoblastic leukemia, which was also correlated with disease prognosis, while others^7^ have not.

The objective of the present study was to analyze p15 gene inactivation by homologous deletion and point mutations in Brazilian children with acute lymphoblastic leukemia.

## SERIES AND METHODS

A total of 106 pediatric patients with acute lymphoblastic leukemia were admitted for treatment to the Pediatric Clinic of the Faculdade de Medicina de Ribeirão Preto, Universidade de São Paulo, between January 1991 and June 1999. Eighty-three patients were eligible for the study and 23 were excluded due to the lack or poor quality of stored DNA. The diagnosis was based on morphological analysis and immunophenotyping with monoclonal antibodies via flow cytometry. All patients were classified and treated according to the protocols proposed by the Brazilian Group for the Treatment of Childhood Leukemias (GBTLI 85/93).^8^ The project was approved by the Research Ethics Committee of the University Hospital (case no. 3761/98). This was a retrospective study of 87 DNA samples extracted from cells aspirated from the bone marrow of 83 children and adolescents with acute lymphoblastic leukemia (83 samples obtained at diagnosis and four also during relapse), and two cerebrospinal fluid samples from two cases with isolated central nervous system relapse.

The p15 gene inactivation was detected by the polymerase chain reaction^9^ for the identification of deletions, confirmed by multiplex polymerase chain reaction using the primers shown below and primers for beta-globin, and complemented by polymerase chain reaction/single-strand conformational polymorphism^10^ for the screening of point mutations. DNA was extracted by the phenol-chloroform method^11^ and quantified, adjusting the concentration to 0.1 μg/ml. The following primers corresponding to the two exons of the p15 gene were used:^5,12^

- exon 1/primer 1:
  5’-AAGAGTGTCGTTAAGTTTACG-3’
- exon 1/primer 2:
  5’-ACATCGGCGATCTAGGTTCCA-3’
- exon 2/primer 1:
  5’-GGGTGGGAAATTGGGTAAG-3’
- exon 2/primer 2:
  5’-TGAGTTTAACCTGAAGGTGG-3’

### Statistical analysis

The 87 bone marrow samples studied corresponded to all the samples stored up to 1998 whose DNA was still viable, and the two cerebrospinal fluid samples belonged to two patients who presented isolated central nervous system relapse during the study period.

The results were compared using the Student t test when the variances were homogeneous and the Bartlett and Mann-Whitney tests when the variances were non-homogenous. Categorical variables were analyzed via the chi-square test. The level of significance was set at 95% (p < 0.05), with a cutoff date of September 1999.

## RESULTS

The 87 bone marrow samples and the two cerebrospinal fluid samples were submitted to amplification by the polymerase chain reaction. The p15 gene was present in 68 bone marrow samples and one cerebrospinal fluid sample. These samples were tested via single-strand conformational polymorphism and did not show any alteration in the electrophoretic migration pattern on polyacrylamide gels for any of the exons analyzed.

Fifteen samples (including one of cerebro-spinal fluid) originating from 15 patients that were not amplified after three replications were tested via the multiplex polymerase chain reaction using the beta-globin primers, which confirmed the deletions. All 15 cases were deletions of exon 2. Eleven deletions were detected in boys and four in girls.

Twenty-two of the 68 patients without deletions and seven of the 15 patients with deletions died during the study period. (33.8 versus 46.6%).

Table 1 lists the classic laboratory parameters used for the definition of risk groups according to the occurrence of the exon 2 deletion. The occurrence of p15 gene deletion was also studied with respect to age and number of white cells at diagnosis, and with respect to immunophenotype, risk group, survival time (total and unfavorable event-free survival) and present situation, and no correlation was detected.

**Table 1:** Relationship between p15 gene deletion and stratifying risk criteria

			With deletion	Without deletion
Gender	Male		11	43
	Female		4	25
Age at diagnosis (months)	> 12		0	3
	12 to 120		11	52
	< 120		4	13
White blood cells at diagnosis (/dl)	> 10,000		5	28
	10,000 to 50,000		7	24
	< 50,000		3	16
Immunophenotype	Pre-B	without myeloid markers	6	32
		1 myeloid marker	2	10
		2 myeloid markers	2	1
	Early pre-B	without myeloid markers	1	3
		1 myeloid marker	0	2
	T	CD10-	3	9
		CD10+	0	2
	B		1	2
	Biphenotypic		0	3
	Unknown		0	4
Risk	Basic	WBC < 10,000/dl	6	14
		WBC ≥ 10,000/dl	3	14
	High		6	40
Total survival (months)	< 12		5	18
	12 to 35		4	12
	36 to 60		2	17
	> 60		4	21
Event-free survival (months)	< 12		6	21
	12 to 35		4	18
	36 to 60		3	12
	> 60		2	17
Present situation	Not treated		3	25
	Under treatment		5	21
	Death		7	22

*WBC = white blood cells.*

## DISCUSSION

Since the mid-1980s, the 9p21-22 region has been recognized as an important site of alterations in patients with cancer, especially children. Through the identification of new tumor suppressor genes by analysis of non-random deletions in this region,^13^ and through the analysis of the p15, p16 and p19 genes located in this region, the role of their inactivation in the genesis and evolution of lymphohematopoietic neoplasias, especially childhood acute lymphoblastic leukemia, has been studied. Some studies^6,12-16^ have emphasized the importance of p15 inactivation in T-cell acute lymphoblastic leukemia, whereas others^17^ have not found any difference between T-cell and B-cell precursor acute lymphoblastic leukemia. Another aspect is the interdependence of p15 and p16, since some studies have shown homologous deletion of both genes in most acute lymphoblastic leukemia cases,^5,6,14,18,19^ while in others p16 was the only target.^6,20,21^ Furthermore, while deletion seems to be one of the main mechanisms of inactivation for both genes, point mutations may play an important role in p16 inactivation, while epigenetic mechanisms such as hypermethylation are also considered for p15.^6,20^

These studies have opened up prospects for the definition of a diagnostic and/or prognostic gene marker. The p15 gene has been identified by some authors as a tumor suppressor gene that can be correlated with disease prognosis.^5,18,22^ In the present study, deletion of exon 2 was identified in 18% of a sample of Brazilian children, mainly originating from the southeastern region, where there is great ethnic diversity in population groups. This result indicates that p15 inactivation by homologous deletion of exon 2 might represent an important molecular marker for the population studied, as also observed by others.^5,13^ However, in contrast to these other studies, we could not establish an unequivocal association between inactivation and classic risk factors. This finding may be explained by the characteristics of the children studied, considering their ethnic diversity and miscegenation, which differ from those observed in studies carried out in Sweden and Japan.^5,18^ No study carried out in Brazil or Latin America to analyze tumor suppressor genes of the 9p21 region in children with acute lymphoblastic leukemia is available in the literature. Thus, since deletions are not the only mechanism for tumor suppressor genes inactivation, the present findings indicate the need for further multicenter studies investigating epigenetic and gene expression mechanisms in order to understand the role of these genes and their interactions in cell cycle progression and carcinogenesis in Brazilian children with acute lymphoblastic leukemia.
